# Temporal analysis of posts on a Japanese online message board for suicide risk monitoring

**DOI:** 10.1186/s12888-025-07539-z

**Published:** 2025-11-20

**Authors:** Takahiro Arai, Hiroyuki Shinkai, Keita Yamauchi

**Affiliations:** 1 School of Management and Information Sciences, Tama University, 4-1-1 Hijirigaoka, Tama-Shi, Tokyo, 206-0022 Japan; 2 Graduate School of Health Management, Keio University, Kanagawa, Japan; 3 Faculty of Law, Kanagawa University, Kanagawa, Japan

**Keywords:** Japan, Online message board, Temporal patterns, Diurnal variation, Mental health, Suicide, Suicidal ideation, Generalized additive model

## Abstract

**Background:**

Suicide prevention can be significantly enhanced by time-sensitive surveillance using digital data sources like online message board. To inform more effective suicide prevention strategies, this study analyzes the temporal patterns of posts on a Japanese mental health message board—NHK’s “Facing Suicide” website—that may aid in early risk detection.

**Methods:**

We analyzed 63,046 posts from Japan’s national broadcaster (NHK) message board (1 Jan, 2008–31 Mar, 2025), stratified by gender and age (≤19, 20s, 30s, ≥40). Generalized additive models were used to model hourly, weekly, and monthly variations, with time of day included as a spline term. Results are presented as incidence rate ratios (IRRs) with 95% confidence intervals (CIs).

**Results:**

Females contributed 75.5% of posts, and the 20–29 age group was the most active (32.4%). Posting activity consistently peaked around 23:00 across all subgroups. A marked increase was observed among adolescents (≤19 years) in August (males: IRR = 1.30, 95% CI [1.05–1.61]; females: IRR = 1.55, 95% CI [1.43–1.69]), while adults showed decreases in January–February. Weekly patterns varied by subgroup; for instance, males aged 20–29 posted more on Mondays and Tuesdays (IRR = 1.17, 95% CI [1.05–1.30]).

**Conclusions:**

Online message board activity displays predictable temporal cycles with demographic-specific patterns. These findings provide an essential baseline for real-time monitoring systems to detect deviations that may signal elevated suicide risk. The August peak in adolescent posts aligns with back-to-school distress, and the late-night peaks underscore the need to provide 24-hour support services and implement automated multi-layered online intervention strategies directly within message boards. These insights can guide the development of targeted, time-sensitive suicide prevention strategies.

**Supplementary Information:**

The online version contains supplementary material available at 10.1186/s12888-025-07539-z.

## Introduction

Suicide is a serious and complex public health challenge with serious consequences not only for individual lives but also for society as a whole. The establishment of robust surveillance systems is essential for the rapid detection and prevention of suicide, and this is one of the key focus areas of global health policy [1, 2]. Extensive research has documented temporal patterns in suicide, showing that occurrence varies by season, day of the week, and time of day [3, 4].

Such patterns suggest the influence of both biological and environmental factors, with increased occurrence of suicide noted in spring and autumn, on Mondays and in the early morning [5, 6]. In Japan, analysis of national data from 1974 to 2014 revealed significant diurnal and weekly variations in suicide rates [7]. The study found that suicide peaked on Mondays for all age groups, with females and older males having a higher incidence during the day, and young men aged 20–39 years were at highest risk during the late-night hours. Such insights highlight the need to tailor time-sensitive intervention strategies to the time of day when suicide risk is high.

While traditional data sources have provided valuable insights into temporal suicidal tendencies, the need to integrate digital data sources such as internet search queries and social media activities into surveillance activities is being recognized [8–10].

Online behavior can act as an early warning sign for suicidal ideation, providing real-time data that can enhance predictive models [11–14]. Nevertheless, research that explores cyclical patterns and demographic‑specific risks in online environments remains limited.

This study aims to fill this gap by analysing postings on an online message board established by the Japanese public broadcaster “NHK” (NHK message board), investigating the cyclical nature of posting behavior across different demographic backgrounds. By utilising this data, this study seeks to advance the early detection of suicide risk and lay the foundation for more effective prevention strategies tailored to specific temporal and demographic patterns.

## Methods

### Data

Data were extracted from the NHK message board hosted on NHK’s “Facing Suicide” website, where users anonymously discuss mental health concerns, including suicidal ideation [15]. Posts often include expressions of despair (e.g., “I want to die,” “I want to kill myself”), as well as accounts of severe stress in daily life (e.g., at school or work). Accordingly, the posts analyzed in this study include both content related to suicidal ideation and content reflecting high‑stress states. Posts containing inappropriate language (e.g., discriminatory expressions; violations of privacy or honor; threats of harm to self or others) may be withheld in whole or in part. All published content is publicly viewable. When posting, users select a username and optionally indicate gender (recorded as male/female in the platform), age, and place of residence. Timestamps are recorded to the minute.

We analyzed counts of posts irrespective of content.

The dataset comprised 90,900 posts from 1 January 2008 to 31 March 2025. We used gender, age, and timestamp variables and created eight strata by crossing gender (male, female) with age (≤19, 20s, 30s, ≥40).

Posts from 1 January 2008 to 31 December 2012 (*n* = 137) were excluded because they were structured as responses to specific questions rather than open-ended posts. In addition, cases where the gender or age of the poster was unspecified (*n* = 27,717) were excluded. After applying these criteria, final analytic sample comprised 63,046 posts.

The daily trend in posting increased from 1 April 2019 onward (Additional file 1). This increase suggests that the NHK message board was not widely known before this date. Therefore, we performed additional analysis with a time span from 01 April 2019 to 31 March 2025.

To account for COVID‑19, we also examined whether key trends held during a pandemic period defined as 1 February 2020–31 January 2023—spanning the month of the first confirmed COVID‑19 fatality in Japan [16] through the month when the government announced its policy to reclassify COVID‑19 as a “Class 5” infectious disease [17].

### Statistical analysis

We fitted generalized additive models (GAMs) to model variation in post counts, as GAMs flexibly capture non‑linear relationships, including cyclical patterns in time‑series data [18]. Following prior work on suicide‑related posting patterns on Reddit [19], the outcome was the number of posts by time of day. Explanatory variables included month and day‑of‑week dummies; time of day was modeled with a spline term. We specified a Poisson distribution with a log link, so that coefficients are interpretable as log incidence rate ratios (IRRs). In our generalized additive model, Sunday was used as the reference category for the day of the week, and March was used as the reference category for the month.

We conducted an additional sensitivity analysis to adjust for calendar effects in our monthly-level GAM. Specifically, we incorporated two adjustments: first, we included log of the number of days in the month as an offset term to account for varying month lengths. Second, we included the year as a categorical covariate to control for long-term, inter-annual variations.

Analyses were conducted in R (version 4.3.0) using the mgcv package. Two‑sided *p* < 0.05 was considered statistical significance.

This study was reviewed and approved by the Education Research Promotion Committee, Faculty of Management and Information, Tama University (Approval No. ERPC_R7_001). All procedures were conducted in accordance with the principles of the Declaration of Helsinki. As the study analyzed de-identified, publicly available data, the requirement for individual informed consent was waived by the committee.

## Results

A total of 63,046 posts from the NHK message board were analyzed (Table 1).

**Table 1:** Number of posts and relative frequency by gender, age, month, day of the week and time of day (hour)

Category	Variable	N	(%)
Gender			
	Males	15,444	(24.5)
	Females	47,602	(75.5)
Age			
	≤19 y	12,805	(20.3)
	20–29 y	20,418	(32.4)
	30–39 y	11,830	(18.8)
	≥40 y	17,993	(28.5)
Month			
	Jan	4,774	(7.6)
	Feb	4,802	(7.6)
	Mar	5,532	(8.8)
	Apr	5,241	(8.3)
	May	5,635	(8.9)
	Jun	4,818	(7.6)
	Jul	5,381	(8.5)
	Aug	5,994	(9.5)
	Sep	5,526	(8.8)
	Oct	5,209	(8.3)
	Nov	5,273	(8.4)
	Dec	4,861	(7.7)
Day of the week			
	Sun	9,364	(14.9)
	Mon	9,748	(15.5)
	Tue	9,174	(14.6)
	Wed	9,264	(14.7)
	Thu	9,308	(14.8)
	Fri	8,138	(12.9)
	Sat	8,050	(12.8)
Time of day (Hour)			
	00:00	5,333	(8.5)
	01:00	4,077	(6.5)
	02:00	2,840	(4.5)
	03:00	1,993	(3.2)
	04:00	1,309	(2.1)
	05:00	963	(1.5)
	06:00	881	(1.4)
	07:00	1,071	(1.7)
	08:00	1,432	(2.3)
	09:00	1,423	(2.3)
	10:00	1,622	(2.6)
	11:00	1,892	(3.0)
	12:00	1,945	(3.1)
	13:00	2,009	(3.2)
	14:00	2,049	(3.3)
	15:00	2,077	(3.3)
	16:00	2,261	(3.6)
	17:00	2,595	(4.1)
	18:00	2,715	(4.3)
	19:00	3,038	(4.8)
	20:00	3,688	(5.9)
	21:00	4,707	(7.5)
	22:00	5,355	(8.5)
	23:00	5,771	(9.2)

Females were the predominant contributors, accounting for 75.5% (*n* = 47,602) of posts. The largest age group was 20–29 years (32.4%, *n* = 20,418), followed by those aged 40 and over (28.5%) and 19 and under (20.3%).

Monthly postings peaked in August (9.5%, *n* = 5,994), with high activity also observed in May (8.9%) and July (8.5%). Weekly patterns showed a peak on Monday (15.5%, *n* = 9,748) and Sunday (14.9%), with the lowest activity on Friday and Saturday. A clear diurnal pattern was observed, with posting activity peaking late at night. The highest number of posts occurred at 23:00 (9.2%, *n* = 5,771), followed by 22:00 (8.5%, *n* = 5,355) and 00:00 (midnight) (8.5%, *n* = 5,333). Conversely, the early-morning hours (05:00–07:00) showed the lowest activity.

**Fig. 1:**
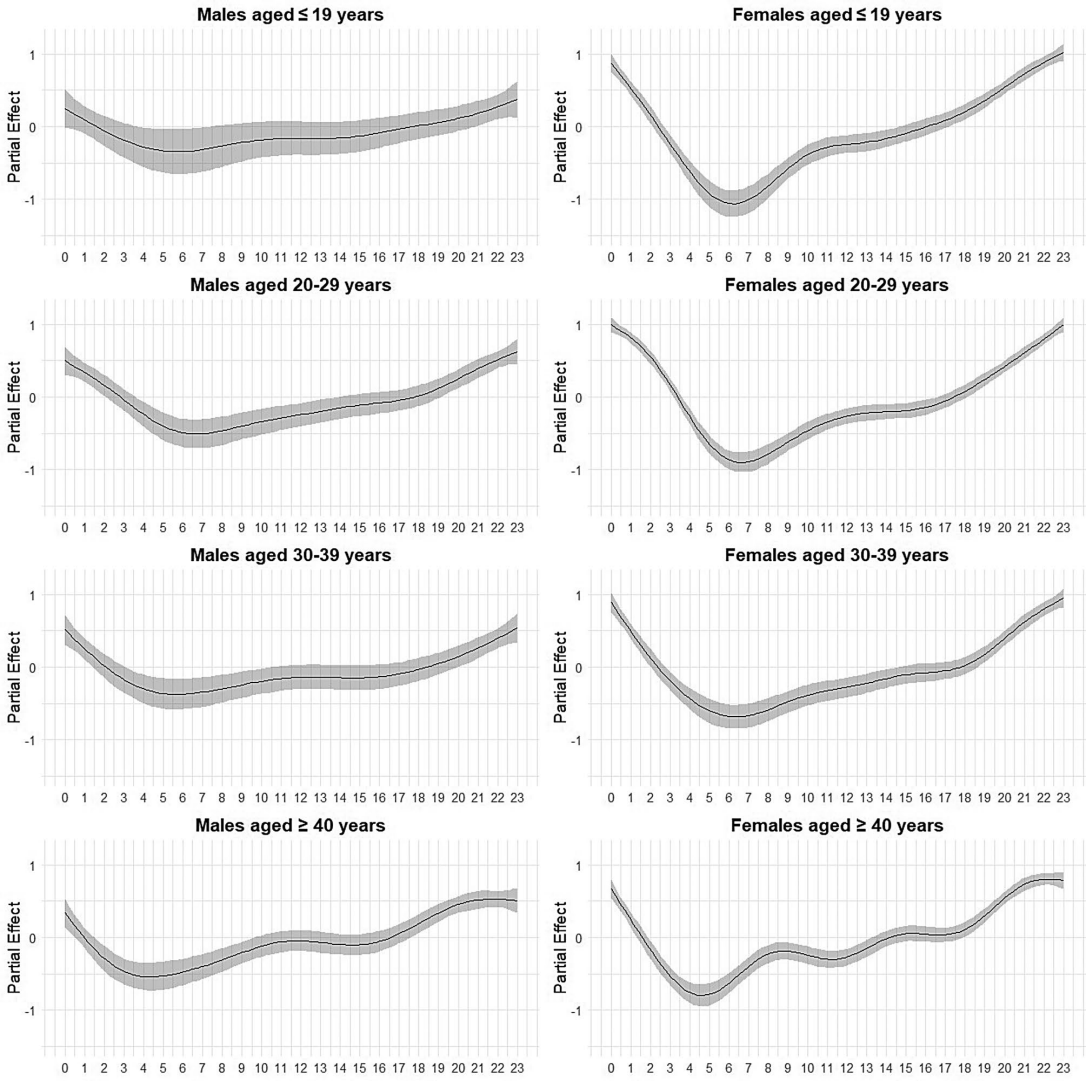
Spline estimates for time effects by gender and age group. This figure shows the estimated effects and 95% confidence intervals for time-of-day spline terms from a generalized additive model (GAM). The analysis is segmented by gender and age group (≤19, 20s, 30s, ≥40). Estimates reflect the smoothed influence of time-of-day variations on the response variable

**Fig. 2:**
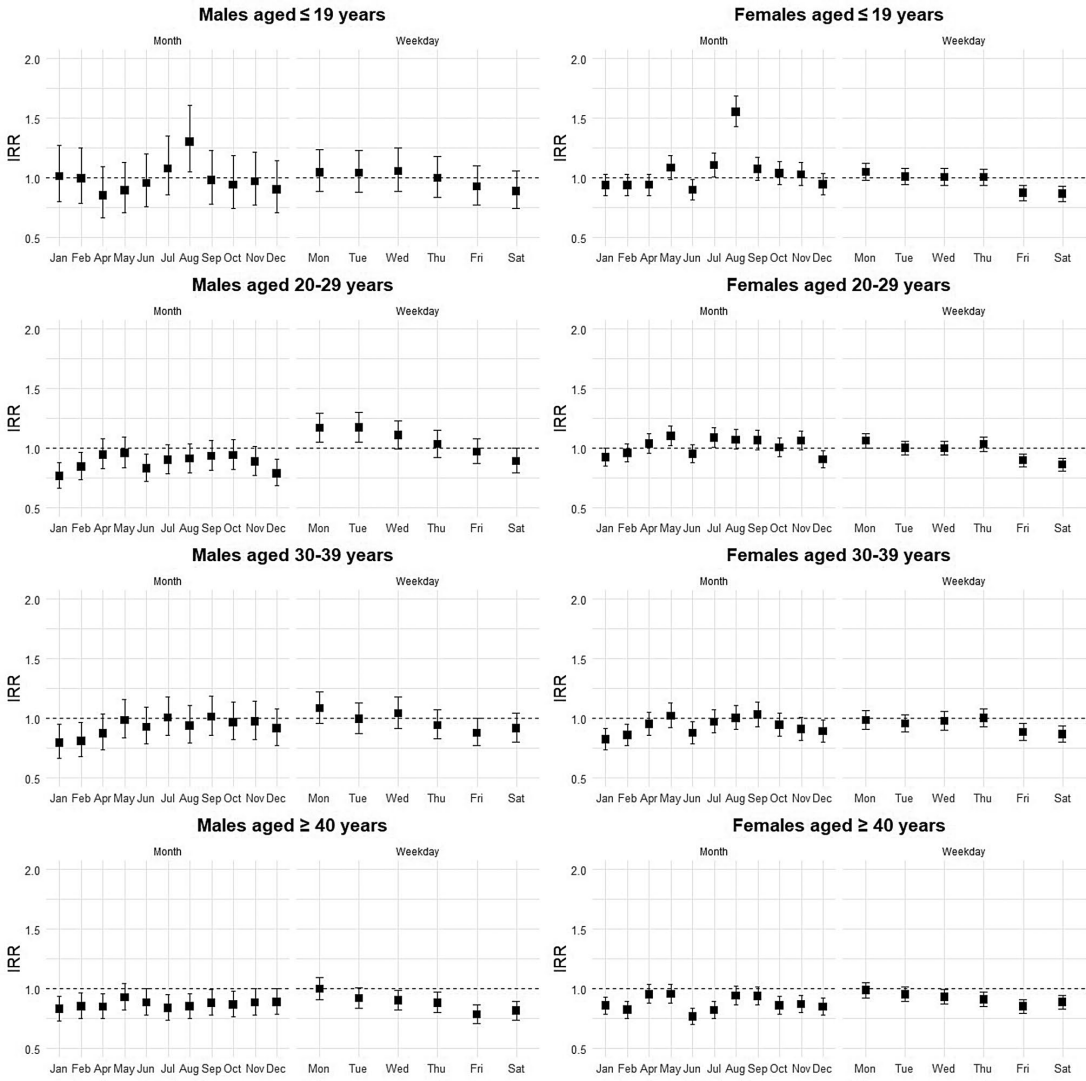
Estimates for monthly and Weekday dummies by gender and age group. This figure shows estimated coefficients and 95% confidence intervals for monthly and weekly dummies from a generalized additive model (GAM), stratified by gender and age group (≤19, 20s, 30s, ≥40). The reference categories are March for months and Sunday for day of the week. The results are presented as incidence rate ratios (IRRs). Positive estimates indicate higher values relative to the reference, while negative estimates indicate lower values. Confidence intervals excluding 1.0 denote statistical significance at the 5% level

Figures 1 and 2 show the results of the GAM analyses, modeling the variation in posting frequency.

Figure 1 shows the smooth term for time of day, confirming diurnal variation. Across subgroups, activity was minimal in the early morning (approximately 03:00–08:00), rose over the day, and peaked around midnight. This pattern was consistent across age and gender strata.

Figure 2 shows monthly and weekly effects by gender and age, presented as IRRs (IRR = exp(β)).

August saw a significant surge in posts among young people, males ≤19 years (IRR = 1.30, 95% CI [1.05, 1.61]) and females ≤19 years (IRR = 1.55, 95% CI [1.43, 1.69])

Conversely, January and February showed significant decreases across several adult subgroups. This included males aged 20–29 (Jan: IRR = 0.76, 95% CI [0.66, 0.88]; Feb: IRR = 0.84, 95% CI [0.73, 0.97]), 30–39 (Jan: IRR = 0.79, 95% CI [0.66, 0.95]; Feb: IRR = 0.81, 95% CI [0.68, 0.96]), and ≥40 (Jan: IRR = 0.83, 95% CI [0.73, 0.94]; Feb: IRR = 0.85, 95% CI [0.75, 0.96]), as well as females aged 30–39 (Jan: IRR = 0.82, 95% CI [0.74, 0.91]; Feb: IRR = 0.86, 95% CI [0.77, 0.95]) and ≥40 (Jan: IRR = 0.83, 95% CI [0.73, 0.94]; Feb: IRR = 0.85, 95% CI [0.75, 0.96]).

In June, significant decreases were observed for males aged 20–29 (IRR = 0.83, 95% CI [0.72, 0.95]) and for females in the ≤19 (IRR = 0.90, 95% CI [0.82, 0.99]), 30–39 (IRR = 0.88, 95% CI [0.79, 0.97]), and ≥40 (IRR = 0.77, 95% CI [0.70, 0.84]) age groups.

In December, declines were seen among both males (IRR = 0.79, 95% CI [0.68, 0.91]) and females (IRR = 0.90, 95% CI [0.84, 0.98]) in the 20–29 age group.

In terms of weekly patterns, there were significantly fewer posts on Fridays and Saturdays compared to Sundays for all age groups of females. This trend was also observed for males aged ≥ 40 years. In contrast, males aged 20–29 posted significantly more on Monday (IRR = 1.17, 95% CI [1.05, 1.30]) and Tuesday (IRR = 1.17, 95% CI [1.05, 1.30]).

The robustness of our main findings was supported by an additional analysis (Additional file 2), which replicated key results, including the August increase for users ≤19 and the weekly patterns for males aged 20–29. A separate analysis of the COVID-19 period (Additional file 3) showed less consistent results, likely due to wider confidence intervals arising from fewer posts, although the August increase for users ≤19 was still observed as a similar trend.

## Discussion

We analyzed 63,046 posts from an online message board dedicated to mental health and suicide‑related topics and identified temporal and demographic patterns relevant to suicide risk surveillance. These findings have implications for digitally enabled prevention strategies and for understanding online behavior during mental health crises.

Demographic patterns revealed that the majority of message board users were female (75.5%). It is important to distinguish the patterns of suicidal ideation and non-fatal suicidal behavior from those of suicide deaths. Epidemiological studies consistently show that males have higher suicide mortality, whereas females exhibit higher rates of suicidal thoughts and deliberate self-harm—a phenomenon often described as the “gender paradox of suicide” [20, 21]. The high proportion of female users in our sample is therefore consistent with the population most affected by suicidal ideation. Importantly, however, self-injurious thoughts and behaviors are well-established predictors of future suicide attempts and deaths [22]. This highlights the public health significance of early detection and intervention at the ideation stage, particularly among young women. The finding that the predominant contributors were female is consistent with previous research suggesting that women are more likely to seek support and express distress [23, 24]. This underscores the importance of creating a digital environment that responds to women’s needs and provides a safe space where mental health issues can be discussed openly [25, 26].

The age distribution shows that users aged 20–29 years are the most active, accounting for 32.4% of the sample. Research reports a significant positive correlation between suicidality and the use of online health message boards, a trend that is particularly pronounced among younger individuals.[27] The high representation of users aged 20–29 years in our data directly aligns with this finding. This age group is not only familiar with digital media but is also often experiencing major life transitions, such as career pressures and changing relationships, which may increase their vulnerability to mental health challenges [28].

The demographic profile of this message board is broadly consistent with the real-world population struggling with suicidal thoughts, underscoring its value for early detection and intervention.

**Fig. 3:**
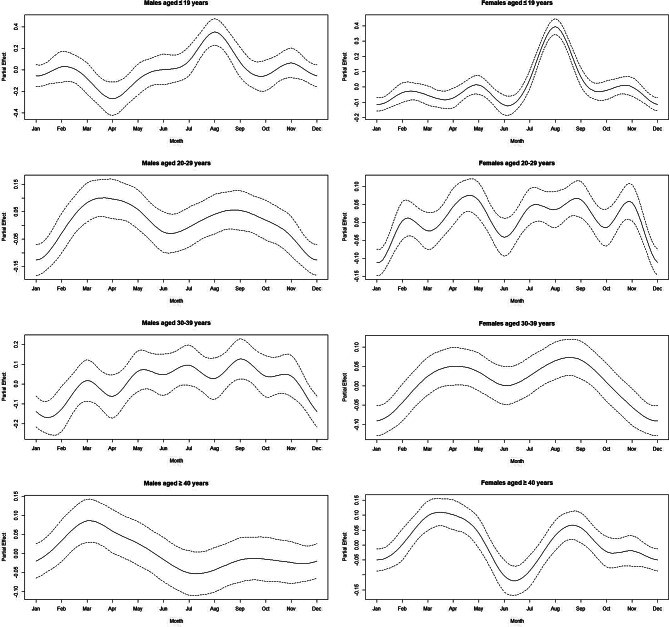
Monthly spline estimates after adjusting for calendar effects. This figure shows the spline estimates and 95% confidence intervals for monthly variations from a generalized additive model (GAM), conducted as a sensitivity analysis. The model adjusts for calendar effects by incorporating the number of days in each month as an offset and including the year as a categorical factor. The analysis is stratified by gender and age group (≤19, 20s, 30s, ≥40)

A key finding was the pronounced August increase in online posts among adolescents (≤19 years) of both genders. After adjusting for calendar effects, the overall monthly patterns remained highly consistent (Fig. 3). The pronounced peak in August, particularly among individuals aged ≤ 19 years, was clearly observed, indicating that this seasonal fluctuation is robust.

This timing coincides with the end of Japan’s summer vacation, when anticipatory stress about returning to school may intensify. Consistent with prior evidence, Google search queries related to school avoidance (e.g., “I do not want to go to school”) also rise in late August [29]. The convergence of these signals suggests that August postings may be an early indicator of the post–summer-break increase in student suicides observed in early September [30]. A plausible psychosocial explanation is the “broken-promise effect” [31]. This term describes the let-down felt when the relief of the vacation ends and is replaced by worries about returning to school. In this view, the message board activity captures this pre-back-to-school distress. Consequently, the NHK message board data may provide important insights into the mental health state of students and could potentially be utilized as a supplementary tool for suicide risk surveillance related to school life.

An increase in postings was observed among females in their 20s during May and July. The May peak may be partly related to the phenomenon colloquially known in Japan as “May illness,” in which fatigue, low motivation, and mild depressive symptoms tend to emerge after the excitement of a new academic or work year subsides [32]. This condition is not specific to young women, but may manifest more prominently in certain groups depending on social expectations and roles.

The slight increase in posts in July can be better understood by considering at the drop in the month before, June. A possible reason for this drop in June is the protective effect of increased sunlight and higher temperature. Japan’s summer solstice is in June, and the longer daylight hours and higher temperature are known to ease symptoms of Seasonal Affective Disorder (SAD) [33, 34]. June also coincides with Japan’s rainy season. While inclement weather is often considered a risk factor for mood, it is also plausible that for introverted individuals, the resulting decrease in outdoor activities may reduce exposure to external stressors, thus acting as a protective factor. The rise in July may simply reflect a return to baseline levels as these protective effects diminish.

On the other hand, it could also be caused by new stressors in July, such as heat-related sleep disruption [35] or academic stress before summer vacation [36]. It is possible that these factors work in combination, and further investigation is needed to clarify their respective impacts.

Conversely, January and February showed significant decreases in postings in several subgroups, including males in their 20s and 30s and over 40s, and females in their 30s and over 40s. These decreases may be attributed to the positive psychological impact of the New Year as a protective factor by encouraging people to set new goals and feel better [37, 38]. Additionally, the New Year period is often marked by family reunions, which can have positive effects on mental well-being. Culturally significant events may serve as a protective factor against suicidal ideation, as they can strengthen social cohesion with family and friends [39, 40]. On the other hand, the psychological distress that should have been reflected in the posts may have been under-represented in January and February, when people were preparing for the start of new lives, such as employment, transfers or enrolment, and did not have the psychological space to post in online message boards. Distinguishing between a true protective effect and a reporting artifact will be an important task for future research.

The pattern of weekly variation showed increased posting on Monday (IRR = 1.17, 95% CI [1.05, 1.30]) and Tuesday (IRR = 1.17, 95% CI [1.05, 1.30]) among males aged 20–29 years. The results supported these previous studies, as it is known that suicide rates increase at the beginning of the week due to the ‘Blue Monday effect’ [41, 42].

The peak of diurnal variation was at midnight, from 22:00 to around 24:00, for all subgroups. These late-night peaks suggest that they may have been brought about by groups with high levels of loneliness and isolation, highlighting the need for mental health resources to be available 24 hours a day [43]. However, providing 24-hour human-staffed services may not be practical. A more feasible and proactive approach would be to implement automated, multi-layered online intervention strategies directly within the message board.

For instance, the system could use keyword detection to automatically direct high-risk users to immediate resources such as a confidential crisis chat service or a guide to nearby mental health services. As a complementary, lower-intensity intervention, the system could also direct users to evidence-based self-coping tools. Such strategies would create a more scalable and immediately responsive support environment.

Some existing support services are only available during the hours between morning and evening, leaving a critical gap at night, when users feel most vulnerable. Expanding support services to cover these hours could help care for people with poor mental health.

Beyond the environmental factors discussed, these temporal patterns may also interact with biological mechanisms. For example, growing evidence suggests that biological rhythms, such as fluctuations in systemic inflammation, modulate vulnerability to suicidality [44]. Integrating this biopsychosocial perspective is worth considering in future research.

These results are not necessarily in line with existing studies. Existing studies on forum-style social media platforms such as Reddit indicate a peak posting time of 5:00 a.m. in the early morning, which differs from the results of our analysis [19]. This may be due to differences in user characteristics or platform structures used in existing studies.

Our study used data from NHK’s online message board, which likely attracts a diverse and representative audience. Compared to other media, which tend to be skewed towards younger age groups, NHK’s message boards appear to be used by a broader general audience. Therefore, it is likely that our data more reflected the wide variety population with suicidal ideation compared to online communities with specific user groups.

Message board-based data, such as that from NHK’s platform, typically consist of longer, more structured narratives in which users articulate their thoughts and experiences in detail. In contrast, general social media platforms often contain shorter, more impulsive posts that include slang or meme-like expressions, which can introduce noise into textual analyses. Consequently, the NHK message board data likely represent a clearer, more deliberate expression of suicidal ideation [45].

## Limitations

The present study has several limitations.

First, the gender and age data are self-reported and may not be accurate. Since users can freely select these attributes, their veracity cannot be confirmed. This may result in misclassification, which could bias our subgroup analyses by, for example, over- or underestimating the posting volume of certain demographic groups.

Second, this study does not take into account user-specific information, such as the geographical location of the user or whether the contributor is a repeat user. It was not possible to obtain unique user information from the data used in this research. Consequently, it is possible that observed peaks in posting activity do not reflect an increase in the number of distressed individuals, but rather repeated posts from a smaller number of highly active users. Investigating these factors in future studies would help to gain more insight into behavioral patterns associated with suicide risk.

Third, this study did not analyze the content of the posts. Analysing the textual content could quickly capture the nature of discussions related to suicide and could provide advantages for understanding and addressing suicidal ideation in real time [46]. Future research should incorporate textual analysis to identify the specific stressors (e.g., academic pressure, loneliness, bullying) that underlie the statistical patterns found in this study.

Fourth, our study was conducted using data from open-access noticeboards and did not capture discussions that occurred in closed communities or on the dark web. On these platforms, where barriers to access are high, conversations may be taking place that promote suicidal behavior and cannot be easily monitored using traditional methods [47, 48]. To develop a comprehensive monitoring system for suicide risk, we should aim to integrate data from a wide range of online sources across cyberspace. Such an approach would allow emerging trends and high-risk behaviors to be more clearly identified and more effectively addressed.

## Conclusion

This study analyzed over 63,000 posts on the NHK message board, highlighting the potential of such platforms for suicide risk surveillance. The primary contribution of this research is the clarification of predictable baseline patterns of online expressions of suicidal ideation; we have identified the typical yearly, weekly, and daily rhythms in which different demographic groups disclose distress.

This baseline understanding is the essential foundation for building a real-time monitoring system. Such a system would function by detecting significant deviations from these established patterns, thereby signaling a potential surge in suicide risk. For instance, it could be crucial for detecting suicide contagion (Werther effect) [49] by tracking how posting patterns change immediately following media reports of a celebrity suicide, allowing for a rapid public health response. Future research, particularly the incorporation of textual content analysis, will further strengthen this system. By analyzing what is being said, not just when, the system could distinguish between general grief and high-risk suicidal ideation, enabling more targeted alerts. Ultimately, this line of research can help transform vast online data from a passive archive into an active tool for predictive suicide prevention.

## Electronic supplementary material

Below is the link to the electronic supplementary material.

Supplementary Material 1

Supplementary Material 2

Supplementary Material 3

## Data Availability

The raw posts analyzed in this study are publicly available from the NHK “Facing Suicide” message board at: https://heart-net.nhk.or.jp/mukiau/message/. For transparency, the aggregated dataset used for the analyses (counts by gender, age, time, and date) and the R code are available from the corresponding author upon reasonable request.

## Abbreviations

NHK: Japan Broadcasting Corporation
GAM: Generalized Additive Model
IRR: Incidence rate ratio
CI: Confidence interval
