# Supplementary material for: Temporal analysis of posts on a Japanese online message board for suicide risk monitoring
Source: BMC Psychiatry. 2025 Nov 20;25:1111. doi: 10.1186/s12888-025-07539-z (PMC12632057; doi:10.1186/s12888-025-07539-z)
Supplement: Supplementary file 1 — Supplementary Material 1 [file 12888_2025_7539_MOESM1_ESM.docx]

**Supplementary Results: Figures S1-2.**


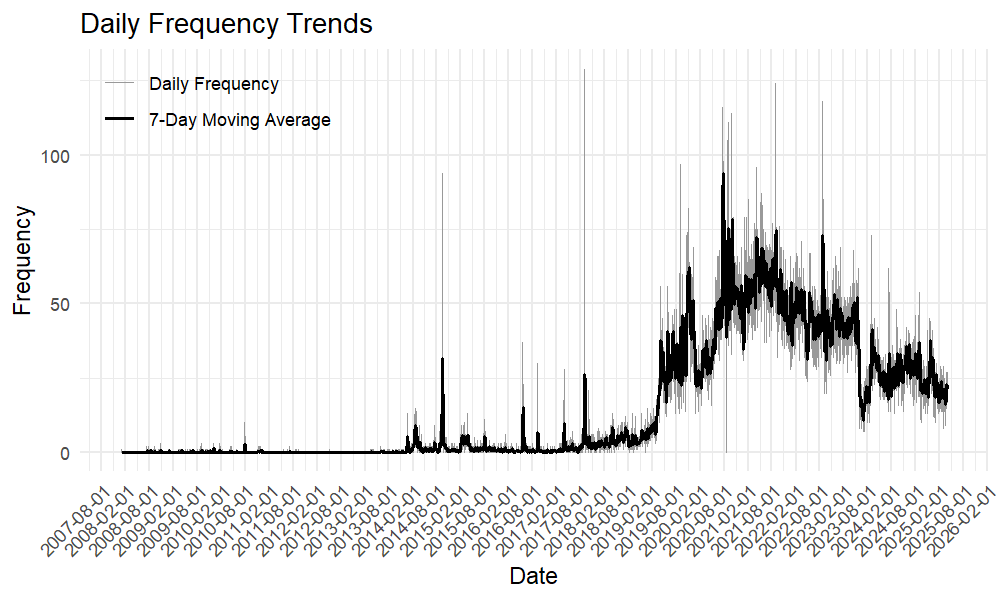


**Figure S1.** Daily (grey line) and 7-day moving average (black line) number of postings on the NHK forum from 1 January 2008 to 31 March 2025.


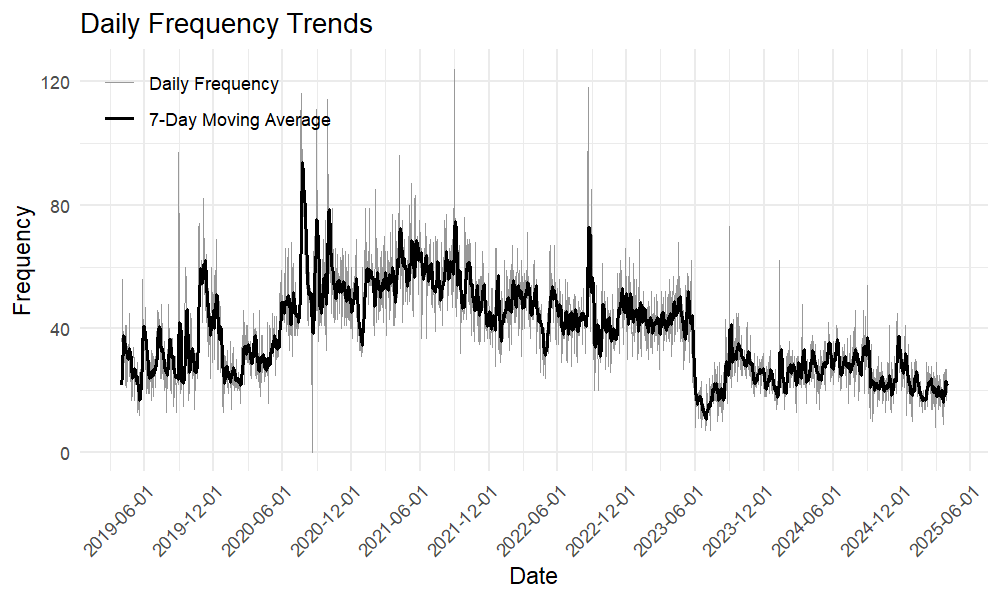
 **Figure S2.** Daily (grey line) and 7-day moving average (black line) number of postings on the NHK forum from 1 April 2019 to 31 March 2025.
