# Supplementary material for: Temporal analysis of posts on a Japanese online message board for suicide risk monitoring
Source: BMC Psychiatry. 2025 Nov 20;25:1111. doi: 10.1186/s12888-025-07539-z (PMC12632057; doi:10.1186/s12888-025-07539-z)
Supplement: Supplementary file 2 — Supplementary Material 2 [file 12888_2025_7539_MOESM2_ESM.docx]

**Supplementary Results: Figures S3 and S4.**


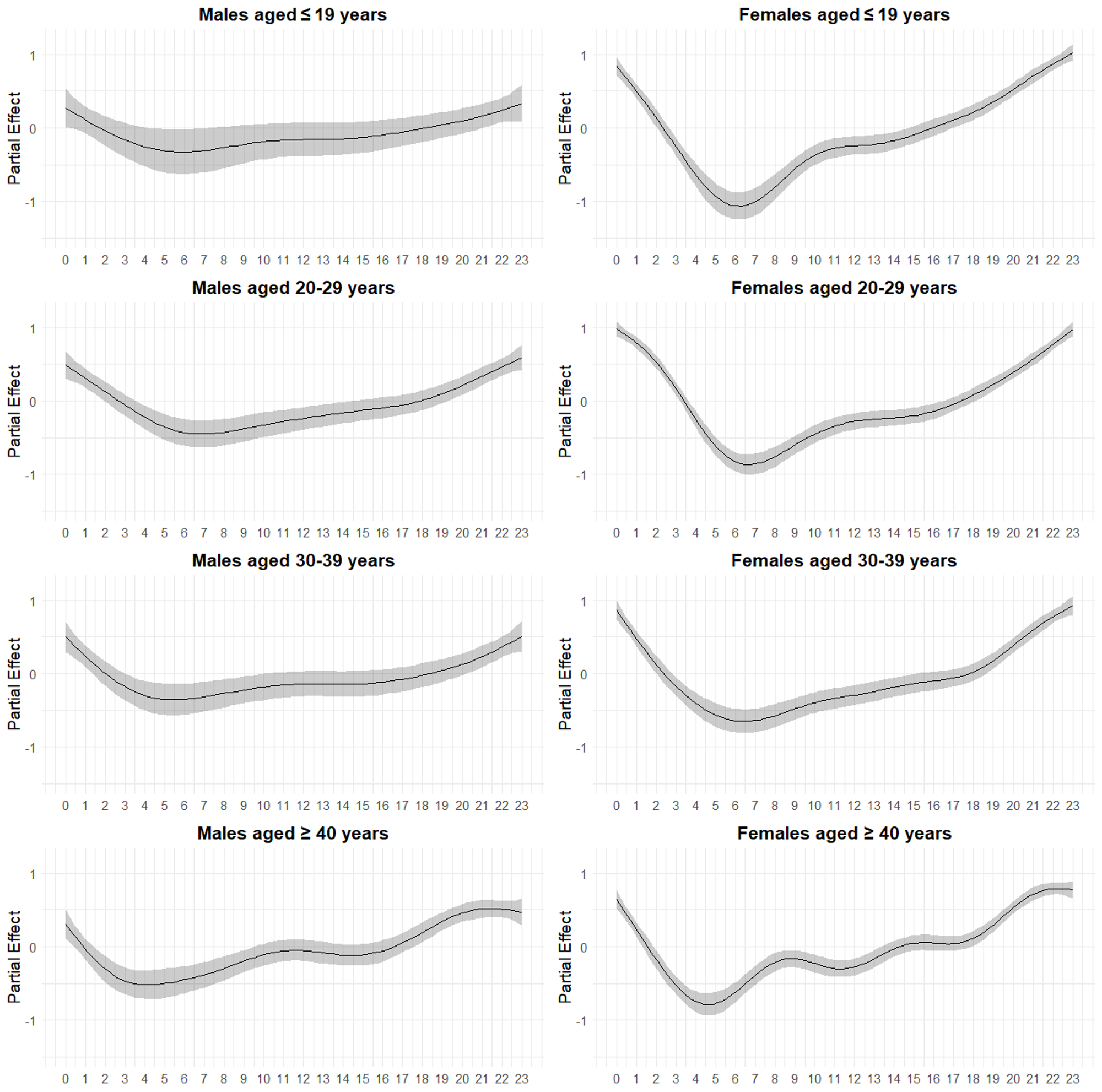


**Figure S3.** Partial effect plots for time-of-day (hour) on posting frequency in the NHK forum by gender and age group (≤19, 20s, 30s, ≥40), April 1, 2019 – March 31, 2025. Plots display GAM spline terms, illustrating relative hourly effects.


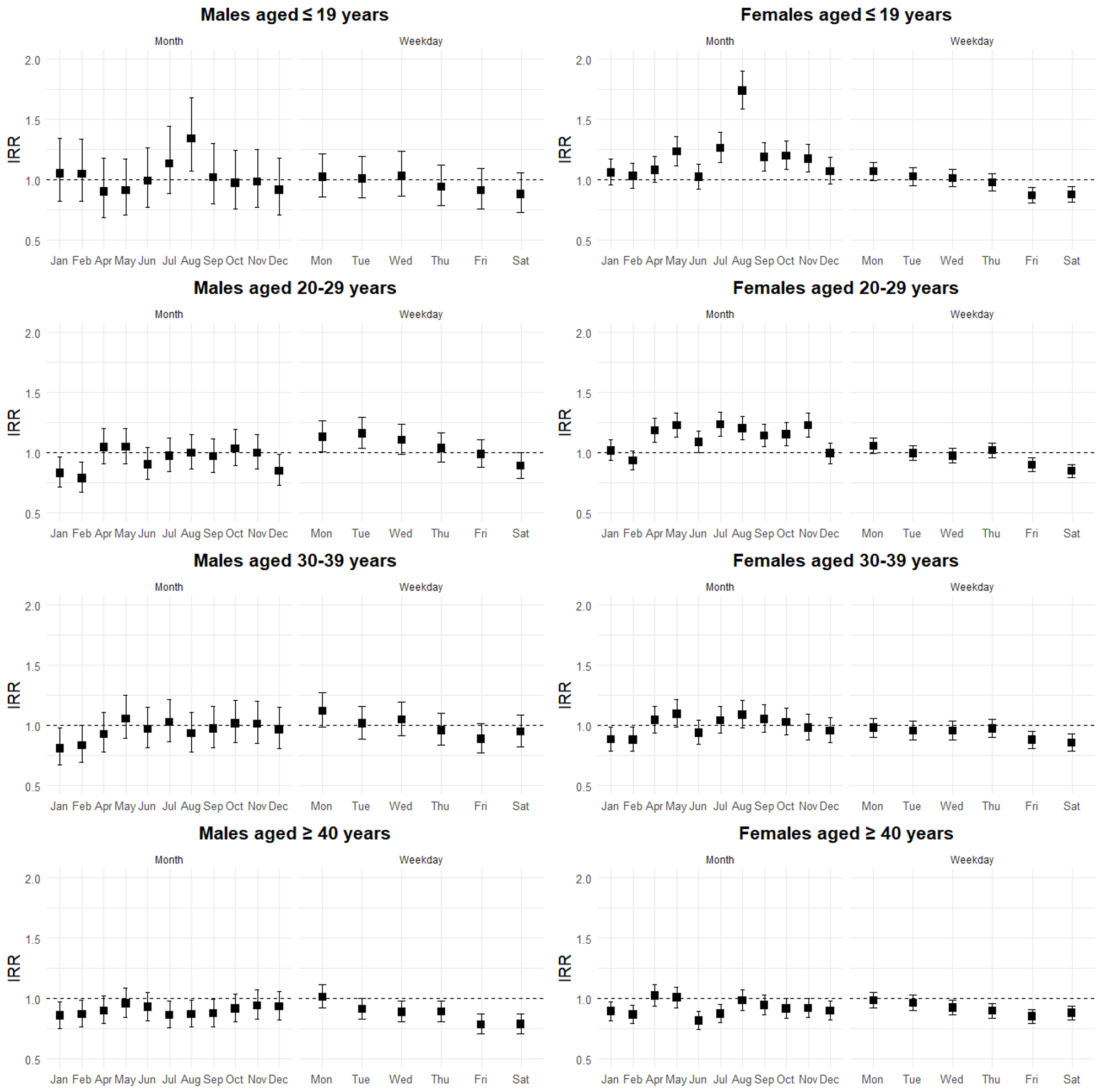


**Figure S4.** Monthly and weekday effects on posting frequency by gender and age group, April 1, 2019 – March 31, 2025. March served as the reference month, and Sunday as the reference day. Results are presented as incidence rate ratios (IRRs).

Summary of supplementary findings:

The time-of-day results were nearly identical to the main analysis.

For males and females, the day-of-week effect did not show substantial differences between the main and additional analyses. These findings reflect a trend in the additional analysis that differs from the main analysis.

These variations suggest that changes during specific periods may influence overall trends in posting behavior. However, the main analysis is positioned as a relatively conservative approach to capture stable trends over the entire period from January 1, 2013, to March 10, 2024. In particular, the coefficients for each month among the overall female showed an increase in the additional analysis, likely reflecting more recent trend shifts.

Therefore, the additional analysis does not substantially alter the conclusions of the main analysis but instead serves a supplementary role in confirming the impact of specific periods.
