# Supplementary material for: Temporal analysis of posts on a Japanese online message board for suicide risk monitoring
Source: BMC Psychiatry. 2025 Nov 20;25:1111. doi: 10.1186/s12888-025-07539-z (PMC12632057; doi:10.1186/s12888-025-07539-z)
Supplement: Supplementary file 3 — Supplementary Material 3 [file 12888_2025_7539_MOESM3_ESM.docx]

**Supplementary Results: Figures S5 and S6.**


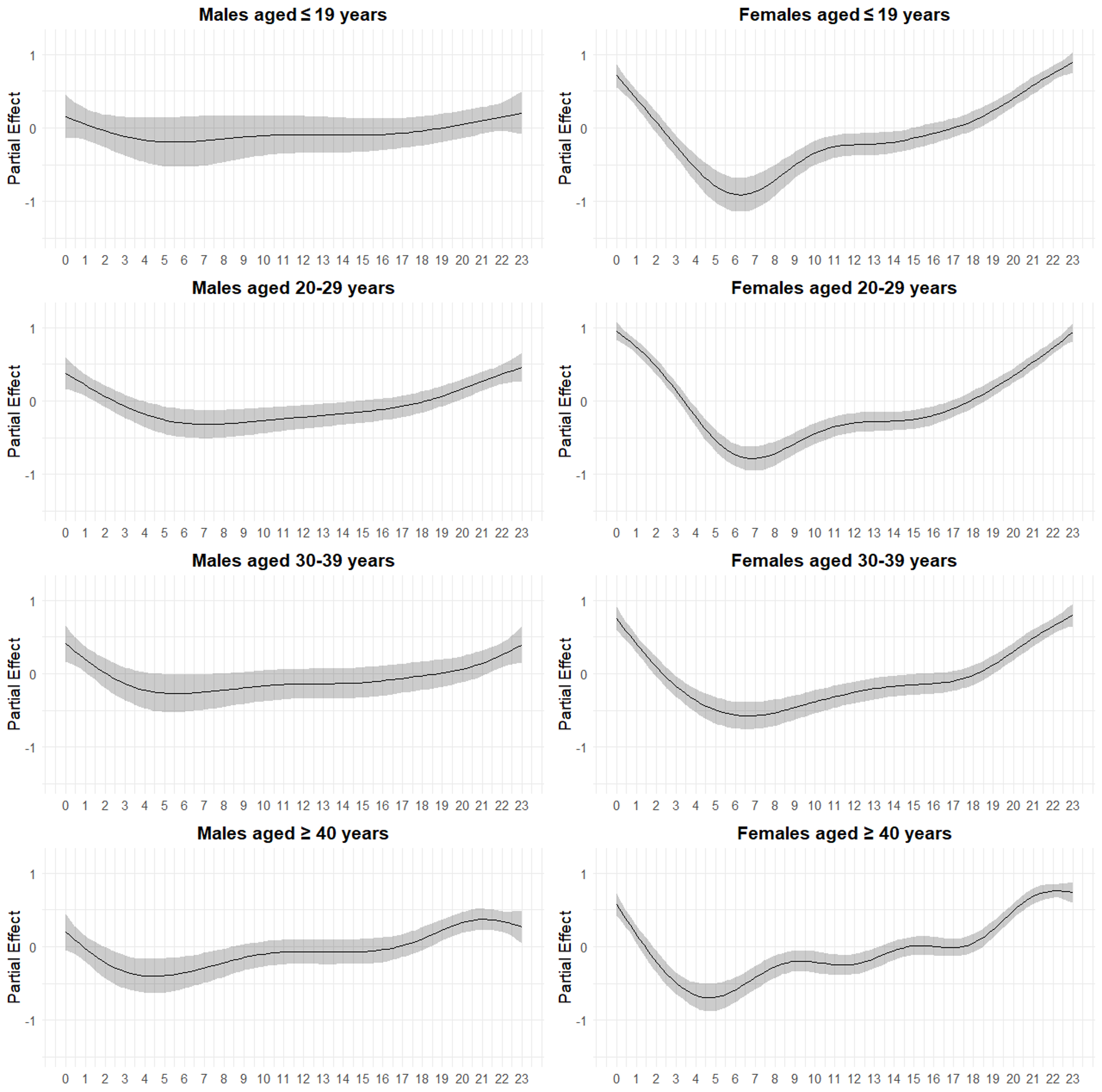


**Figure S5.** Partial effect plots for time-of-day (hour) on posting frequency in the NHK forum by gender and age group (≤19, 20s, 30s, ≥40), 1 February 2020 – 31 January 2023, 2025. Plots display GAM spline terms, illustrating relative hourly effects.


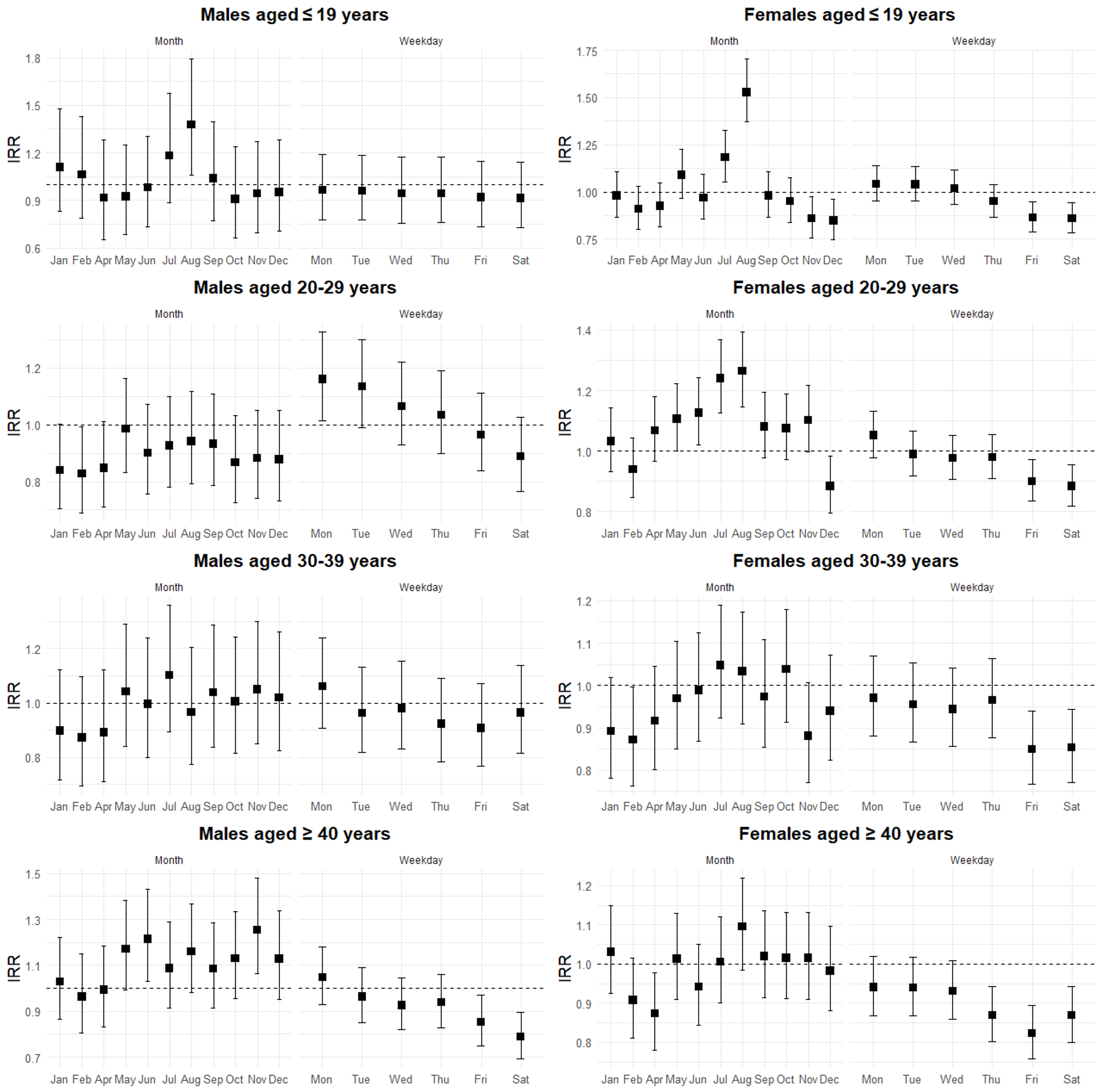


**Figure S6.** Monthly and weekday effects on posting frequency by gender and age group, 1 February 2020 – 31 January 2023. March served as the reference month, and Sunday as the reference day. Results are presented as incidence rate ratios (IRRs).
